# Optical tweezers as an effective tool for spermatozoa isolation from mixed forensic samples

**DOI:** 10.1371/journal.pone.0211810

**Published:** 2019-02-07

**Authors:** Nicole Auka, Michael Valle, Bobby D. Cox, Peter D. Wilkerson, Tracey Dawson Cruz, Joseph E. Reiner, Sarah J. Seashols-Williams

**Affiliations:** 1 Department of Forensic Science, Virginia Commonwealth University, Richmond, Virginia, United States of America; 2 Department of Physics, Virginia Commonwealth University, Richmond, Virginia, United States of America; Duke University Marine Laboratory, UNITED STATES

## Abstract

A single focus optical tweezer is formed when a laser beam is launched through a high numerical aperture immersion objective. This objective focuses the beam down to a diffraction-limited spot, which creates an optical trap where cells suspended in aqueous solutions can be held fixed. Spermatozoa, an often probative cell type in forensic investigations, can be captured inside this optical trap and dragged one by one across millimeter-length distances in order to create a cluster of cells which can be subsequently drawn up into a capillary for collection. Sperm cells are then ejected onto a sterile cover slip, counted, and transferred to a tube for DNA analysis workflow. The objective of this research was to optimize sperm cell collection for maximum DNA yield, and to determine the number of trapped sperm cells necessary to produce a full STR profile. A varying number of sperm cells from both a single-source semen sample and a mock sexual assault sample were isolated utilizing optical tweezers and processed using conventional STR analysis methods. Results demonstrated that approximately 50 trapped spermatozoa were required to obtain a consistently full DNA profile. A complete, single-source DNA profile was also achieved by isolating sperm cells via optical trapping from a mixture of sperm and vaginal epithelial cells. Based on these results, optical tweezers are a viable option for forensic applications such as separation of mixed populations of cells in forensic evidence.

## Introduction

Evidence samples containing biological fluids are frequently encountered in forensic casework, especially with sexual assault cases. Evidence of this nature has the potential to contain a mixed DNA profile, where the combined biological contributions from multiple donors are comingled [1]. Unfortunately, differentiating DNA profiles during downstream mixture interpretation is not a task that is executed with 100% certainty or consistency [2–4]. Thus, for forensic investigations, eliminating mixtures altogether is preferred in order to prevent the problems that accompany interpretation of such data.

In forensic casework, mixtures containing sperm and nonsperm cells are traditionally separated via differential cell lysis prior to DNA extraction [5]. Alternative methods for separation have been the subject of several different approaches over the past 20 years, and include flow cytometry and other antibody-based methods, laser-capture microdissection, and the DEPArray system (Menarini Silicon Biosystems), which utilizes dielectrophoresis technology and cages within a micro-fluidic cartridge to isolate each cell [6–14]. None of these alternate methods have thus far been widely implemented into the forensic community due to challenges such as high hardware costs and extensive additional processing times.

Another approach for separating cells that has been suggested, but not fully explored, is the use of optical tweezers. An optical tweezer is a compact, strongly focused laser beam that uses an immersion objective lens on an inverted microscope to create an optical trap [15]. The optical trap can hold dielectric particles in its center, known as the focal spot. When the laser beam located underneath the particle is moved, the particle is tugged along with it. Optical tweezers allow for the gentle maneuver of objects held in the optical trap, and thus fragile particles can be transferred safely [16].

Optical tweezers have been studied for their practical use in physics, biology, chemistry, nanoscience, and medical science since their development in 1985 [17]. More specifically, optical tweezers from 1064 nm lasers have been used to trap and analyze live sperm cells. *Tadir et al*. [18] and *Nascimetno et al*. [19] studied sperm cell motility under various conditions with optical trapping. Other examples of studies on optically trapped sperm cells can be found as well [20,21]. In 2011, Wang *et al*. implemented optical tweezers on a microfluidic chip for single-cell sorting. Single-trap serial sorting and multi-trap parallel sorting were used to separate yeast cells, based on size, from an aqueous mixture containing both yeast cells and micro-beads. This single-trap serial sorting recovered 97% of the yeast cells present, and 98% of those were sorted correctly [22].

More recently, *Reiner et al*. utilized optical tweezers to capture a single mitochondrion from a human HL-60 cell, and performed multiple rounds of DNA amplification and mitochondrial DNA sequencing to detect the presence of heteroplasmic mitochondrial DNA [23]. Further, an NIJ-funded project by Chakrabarty et al. in 2008 specifically examined the trapping of sperm cells with optical tweezers, where sperm cells were trapped from mock forensic samples and processed for STR analysis [24]. However, due to the less sensitive STR analysis methods employed in 2008, the method required the collection of 400 sperm cells in order to yield sufficient signal for detection of alleles. The large number of cells required made the use of optical tweezers time consuming, tedious and not conducive to inclusion in a forensic analysis workflow. Nevertheless, Chakrabarty’s results exhibited the successful application of optical trapping on forensically relevant samples.

Given the increased sensitivity of modern STR multiplex amplification kits, there is a clear need to reevaluate the use of optical tweezers for separation of mixed cells from sexual assault samples. Further, in order for this to be a viable forensic method, the number of cells required to routinely achieve full STR profiles would need to be empirically determined. The aim of this study was to address these remaining questions regarding the separation and transfer capacity of optical tweezers for isolation of sperm cells from forensically relevant mixtures.

## Methods

### Sample preparation

Semen and vaginal fluid were collected from 5 donors. The Virginia Commonwealth University Human Subjects Institutional Research Board approved the study (HM20002931), and written consent was obtained for all human samples used in the project. Semen was deposited into sterile collection cups and 200 μL aliquoted and frozen at -20°C for future use. Vaginal secretions were collected on swabs by the donors, returned in swab boxes, and stored at room temperature. Semen samples were diluted to 1:5, 1:10, 1:20, and 1:25 using bovine serum albumin (BSA) prepared at 4 mg/mL, as suspension in BSA minimizes the sticking of spermatozoa to glass surfaces. Vaginal swabs were added to 300 μL of ddH_2_0 and the cells were released into solution by agitating at room temperature for approximately 5 minutes. The mock sexual assault sample was prepared by combining equal volumes of vaginal cells released from dry swabs, and semen dilutions ranging from 1:5–1:10, depending on the abundance of sperm as visualized in analysis preparation. Microscope slides were prepared by cementing 0.17 mm thick glass cover slips on the bottom of a standard microscope slide with a 1-cm circular pre-drilled hole. The purpose of this was to make an optically accessible well in which to perform the cellular extractions. The resulting 100 μL well was filled with hexadecane (Sigma Aldrich, St. Louis, MO, USA) which was necessary to eliminate evaporation of the sample. The various cell samples were manually pipetted (0.5 μL) onto the cover slip and the hexadecane allowed the formation of well-defined droplets from which separation and extraction of sperm cells was possible.

### Sperm cell transfer and collection

**Fig 1:**
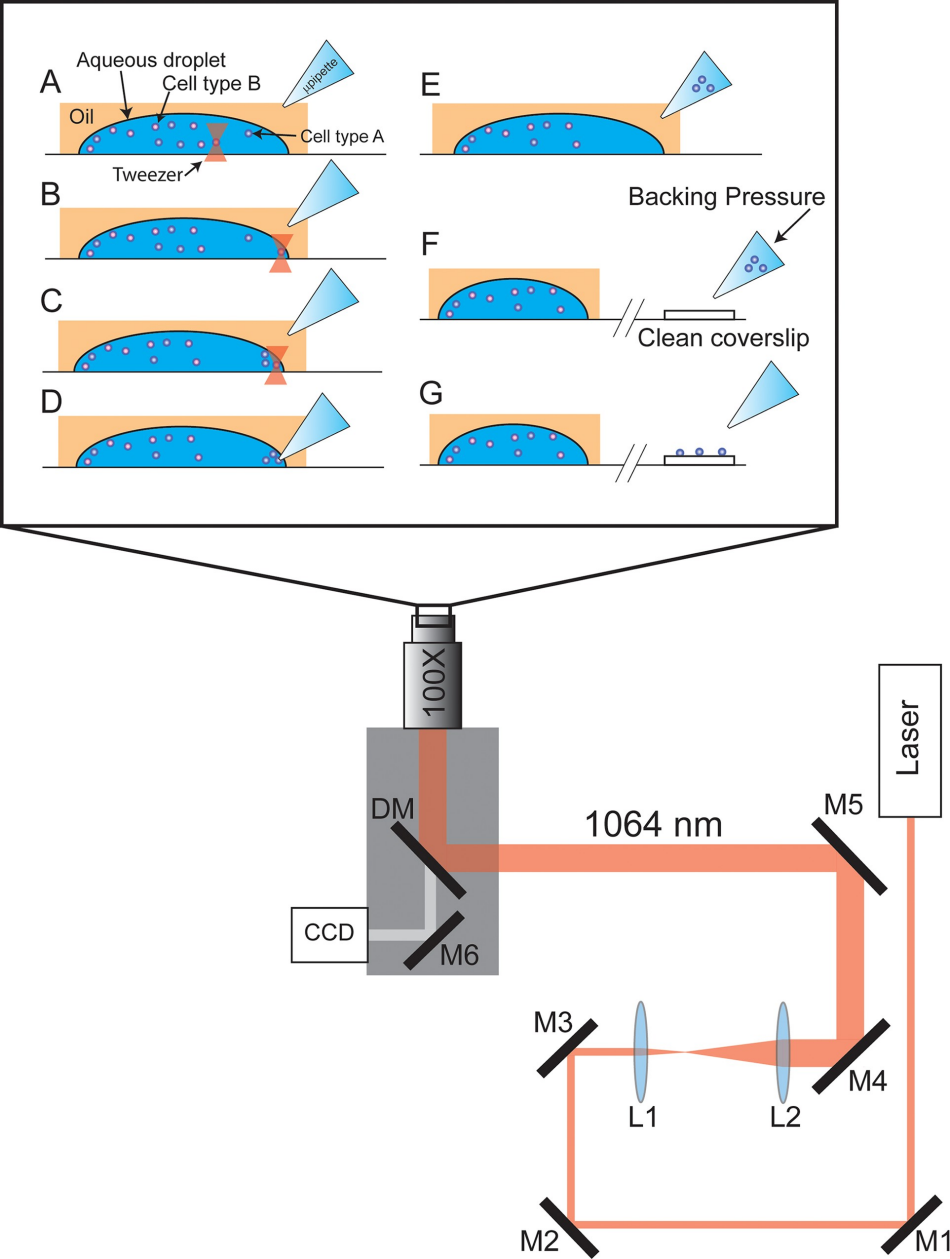
Schematic illustration of the optical tweezer cell separation and collection process. Optical tweezers are formed by aligning the beam from a 1064 nm CW laser into the entry port of an inverted microscope. The beam is expanded with a 2 lens telescope (L1, L2) and reflected off a dichroic mirror (DM) into the back aperture of a 100x NA 1.4 microscope objective. A CCD camera is mounted onto the exit port of the microscope and the experiment is illuminated from above (not shown) with a LED white light source. The zoomed in portion above the microscope illustrates the extraction protocol. **(A)** An aqueous droplet containing a mixture of cells (blue and red circles) is fixed onto a glass coverslip in an oil background (hexadecane). A micropipette tip with a tip diameter of approximately 5–10 microns is positioned nearby. **(B)** Optical tweezers trap a target cell and position it near the edge of the droplet. **(C)** This process is repeated as many times as necessary to separate the desired number of cells. **(D)** The pipette tip is positioned at the edge of the droplet and the capillary force draws the cells up into the tip. **E-G.** The pipette tip is removed from the oil solution and positioned down near a clean coverslip. Backing pressure is applied to eject the trapped cells onto the coverslip.

Fig 1 is a schematic illustration of the cell transfer process using optical tweezers, also referred to throughout this report as “trapping”. The apparatus is constructed on an inverted microscope (AxioObserver D1, Zeiss, Thornwood, NY), that is fixed to an air-floated 3’x4’ vibration isolation table (9100 Series Workstation, Kinetic Systems, Boston, MA). Brightfield illumination is provided by an LED white light source (P/N: LIUCWHA, Thorlabs, Newton, NJ, USA) held fixed about 10 cm above the microscope objective. For the extraction process, the microscope slide was placed onto a motorized microscope stage (P/N: MLS-203, Thorlabs, Newton, NJ, USA) that can be controlled with a joystick (P/N: MJC001, Thorlabs). For trapping, we used an oil immersion 100X magnification objective (Plan-Apochromat NA = 1.4, Zeiss), and a 700 mW, 1064 nm continuous wave (CW) laser (P/N: IRCL-700-1064, CrystaLaser, Reno, NV). The laser was attenuated with an OD1 neutral density filter (P/N: NE10B-B, Thorlabs) and aligned with appropriate optics into the back aperture of the microscope objective. We used a dichroic mirror (P/N: XF2017, Omega Optical, Brattleboro, VT, USA) mounted inside the inverted microscope to reflect the 1064 nm laser light up towards the immersion objective. This mirror transmits a significant portion of the visible spectrum, which allows us to see the extraction protocol with our 1024×768 CCD camera (P/N: DCU223C-BG, Thorlabs) that is mounted onto an exit port of the inverted microscope. Using the joystick, the stage was moved until the laser was positioned over each sperm cell (Fig 1A), and the laser tweezer was then used to capture and transfer the sperm cell to the edge of the droplet (Fig 1B). This process was repeated until a cohort of the desired number of sperm cells was captured (Fig 1C). A borosilicate glass capillary (Sutter OD = 1.0mm, ID = 0.78mm) was placed into a laser-based pipette puller (P-2000, Sutter Instruments, Novato, CA, USA) and a custom heating program (parameters (HEAT = 290, FIL = 004, VEL = 050, DEL = 100) was executed to form a micropipette with an end-tip opening of approximately 10 micrometers. Micropipettes were back-filled with 5 μL of ddH_2_0, mounted to a motorized manipulator (MPC-325, Sutter) and positioned near the cohort of sperm cells just outside the droplet edge (Fig 1D). When the micropipette tip made contact with the droplet, capillary action immediately drew up the tweezed sperm cells (https://www.youtube.com/watch?v=SzxDrWFVcCY) (Fig 1E). After removing the cells, a FemtoJet microinjector (Eppendorf, Hauppauge, NY) was used to eject approximately 0.5 μL of the sperm containing solution onto a sterile cover slip measuring 5 mm in diameter (Thomas Scientific, Swedesboro, NJ, USA) (Fig 1F). After the solution evaporated, the sperm cells were clearly identified and counted to confirm sufficient transfer of cells (Fig 1G).

Afterwards, the cover slip was transferred into a microcentrifuge tube and stored at -20°C for later DNA analysis. Typical extraction time, which starts with the ejection of the aqueous droplet onto the coverslip and ends with the transfer of ca. 50 sperm into the storage centrifuge tube, takes approximately 45 minutes.

### DNA analysis

DNA extraction was performed on each sample using the QIAamp DNA Investigator Kit (Qiagen, Hilden, Germany). The protocol “Isolation of Total DNA from Surface and Buccal Swabs” recommended by the manufacturer was followed with the subsequent deviations: 300 μL of buffer ATL was added to the lysis step along with 20 μL of 1M DTT, carrier RNA was not added to the lysis step, 300 μL of buffer AL was added, and then 150 μL of ethanol was added after the 10-minute incubation period. Each sample was eluted in 30 μL of elution buffer.

DNA quantification was performed on each DNA extract using the Quantifiler Trio DNA Quantification Kit (Applied Biosystems, Foster City, CA) on the ABI Prism 7500 real-time PCR instrument (Applied Biosystems) using half-volume reactions. Data was analyzed using HID Real-Time PCR Analysis Software Version 1.2 (Applied Biosystems). In addition to the sample DNA concentrations, the quantification method was used to evaluate the DNA degradation index. The DNA degradation index (DI) is the ratio of concentration values based on a small DNA target and a large DNA target; DI values >1 indicates DNA degradation [25].

Each extract was concentrated down to approximately 5–8 μL after quantification using the Savant DNA120 SpeedVac concentrator (Thermo Fisher Scientific, Waltham, MA) at a no heat, low drying setting for approximately 15 minutes. Amplification was performed using the AmpFLSTR® Identifiler Plus PCR Amplification Kit (Applied Biosystems) using half-volume reactions on the ProFlex PCR system (Applied Biosystems). Thermal cycling parameters were: 95°C for 11 minutes, 28 cycles of 94°C for 20 seconds and 59°C for 3 minutes, 60°C for 45 minutes, and hold at 4°C. Fragment separation was conducted using the 3130 genetic analyzer (Applied Biosystems) using a 36 cm capillary and POP-4 polymer. STR profiles were analyzed using GeneMapper Software Version 4.1 (Applied Biosystems) with an analytical threshold of 50 relative fluorescent units (RFU). Data collected from each electropherogram included the number of alleles called that were consistent with the known reference profile of the semen sample donor, the number of unknown or unexpected drop-in alleles, and the average STR allele peak height (for samples containing at least 28 sperm cells). Interlocus peak height balance was qualitatively evaluated to assure that alleles across the multilocus profile were appropriate balanced. All quantification and CE data is available in the Supporting Information (S1 Data).

## Results and discussion

**Fig 2:**
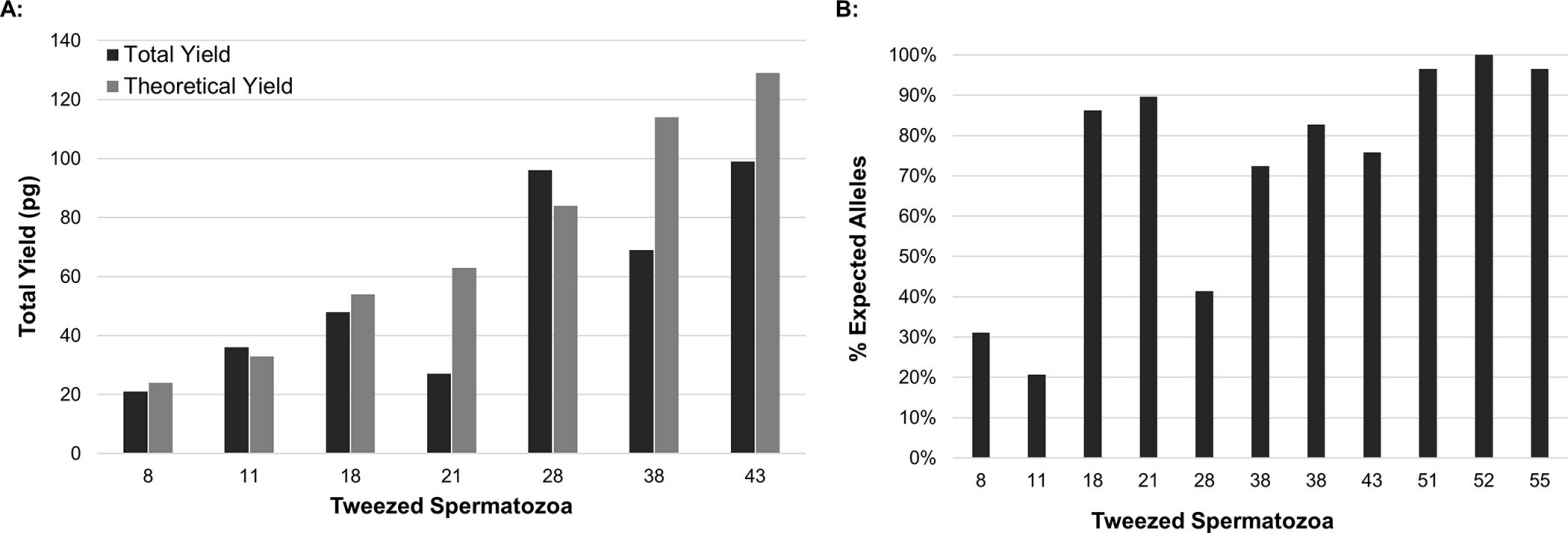
**Total DNA Yields (A) and Percentage of Expected STR Alleles Detected (B) by sperm cell number tweezed.** Cells were tweezed from semen samples. Each bar indicates a single sample.

**Fig 3:**
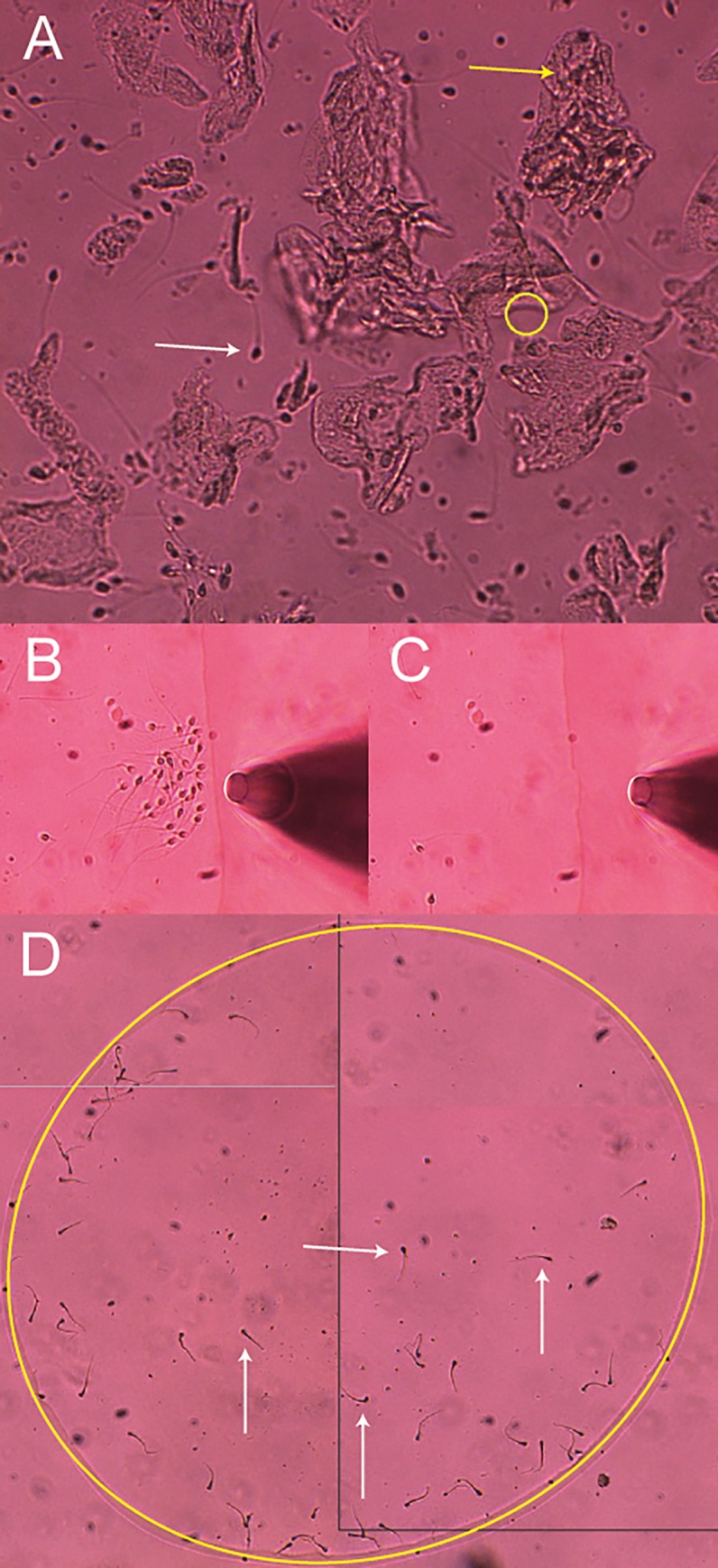
An image gallery showing the cover slip collection method from a mixture sample. **(A)** Mock sexual assault sample containing vaginal epithelial (yellow arrow) and sperm cells (white arrow). The yellow circle indicates the location of the optical tweezer. **(B)** Sperm cells transferred to the right edge of droplet are **(C)** extracted with a pipette tip. **(D)** Sperm cells ejected onto a cover slip. The large yellow oval indicates the edge of the evaporation ring inside which all the sperm cell are localized. Four sperm cell are identified with white arrows. All images captured with a 20x objective.

Initially, sperm cells were tweezed from neat semen samples. Predictably, as the number of extracted sperm cells increased, the total DNA yield (Fig 2A) and percentage of expected STR alleles from the donor (Fig 2B) also increased. Complete or near complete STR profiles were consistently obtained when ≥50 sperm cells were isolated using this method. Mock sexual assault mixtures were then generated using diluted vaginal epithelial cells and sperm cells of 3 unique pairs of donors, and sperm cells were captured and transferred using the same trapping method (Fig 3). In an effort to evaluate cell loss during the transfer process (after trapping), percent recovery was examined. Recovery of the sperm cells from droplet to coverslip was high, ranging from 58–140% (Table 1). However, it should be noted that in one case (Sample 3, replicate C), additional sperm cells were inadvertently pulled into the capillary from the surrounding region of the droplet, resulting in more sperm collected than intended. Nonetheless, using the trapping and cell transfer method described herein, this data suggests that an approximate 15% cell loss should be anticipated going forward.

**Table 1:** Recovery of optical tweezer-separated spermatozoa from mixed vaginal:Sperm samples.

Sample	Replicate	Cells Extracted	Cells Ejected	Percent Recovery
**2**	A	56	52	93%
	B	56	39	70%
	C	77	45	58%
**3**	A	90	58	64%
	B	65	58	89%
	C	53	74	140%
	D	54	46	85%

**Fig 4:**
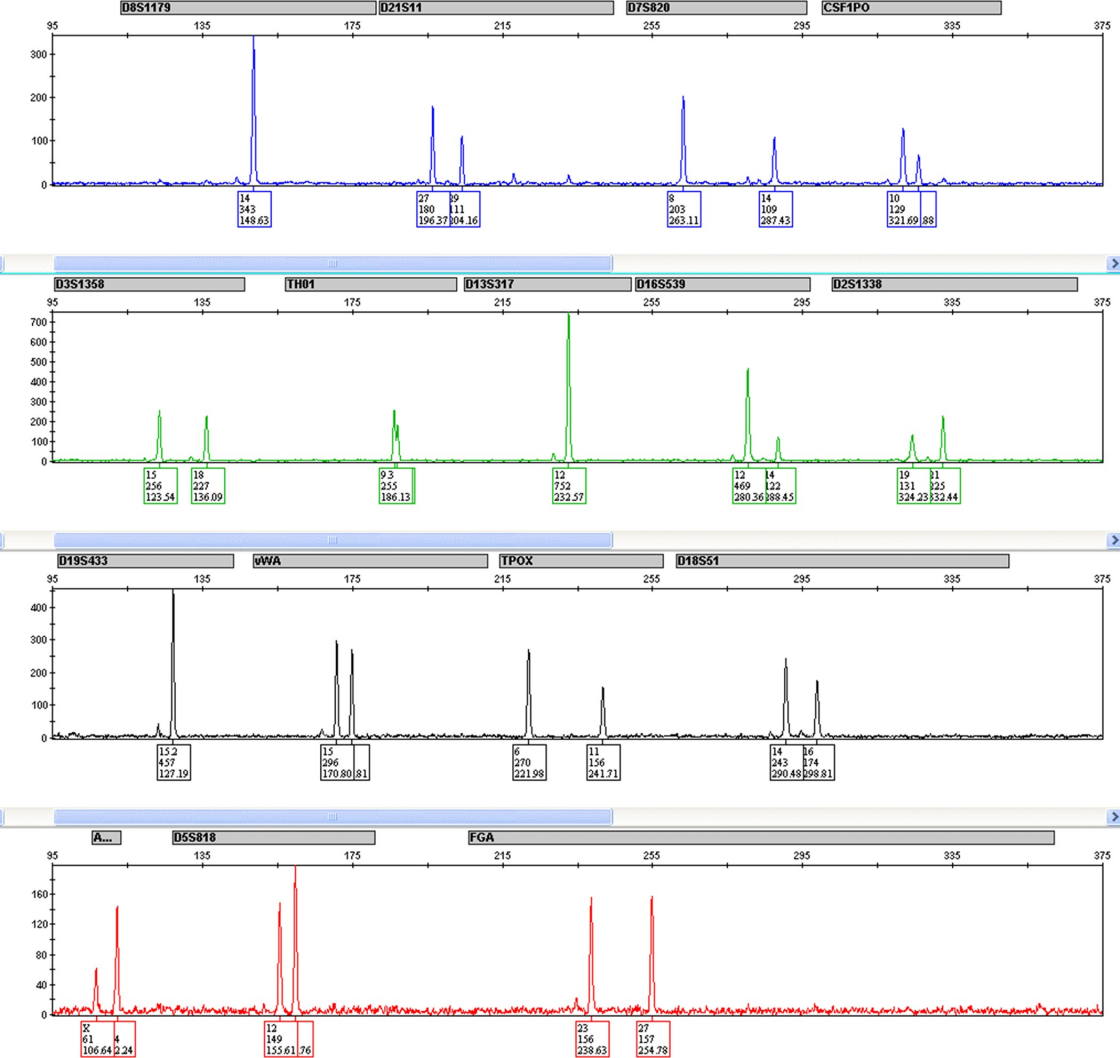
Representative electropherogram of 56 sperm cells tweezed from the mock sexual assault sample. All observed alleles are concordant with the sperm cell donor.

When DNA was purified and STR loci amplified from the mixture samples, we found that using optical tweezers resulted in clean, male-only DNA profiles (Fig 4) from six of the nine mixture samples analyzed (Table 2). As expected given the low DNA yields, mean STR allele peak heights were relatively low (311.5 ± 229.5 RFU), but were consistently distinguishable from background noise (analytical threshold). Most importantly, known alleles from the female contributor to the mixture were only detected in three of nine mixture samples tweezed (Table 2). These data demonstrate that the optical tweezer method detailed herein is capable of consistently providing DNA profiles from the male contributor that can be easily interpreted without complex back-end mixture deconvolution.

**Table 2:** STR results from optical tweezer-separated spermatozoa fractions from mixed vaginal:Sperm samples.

Sample	Replicate	# tweezed cells	Male contributor alleles detected	Percent STR profile (male)	Female contributor alleles detected
1	A	51	26/27	96.3%	0
	B	56	27	100%	0
2	A	52	29	100%	1
	B	39	29	100%	6
	C	45	29	100%	0
3	A	58	26	100%	0
	B	58	26	100%	5
	C	74	26	100%	0
	D	46	26	100%	0

Prior to our testing, there was some concern regarding whether the optical tweezers were damaging the DNA inside the sperm cells. Radiation-induced DNA damage caused by optical tweezers has been examined in other work [16]. According to *Liu et al*. and *Greulich et al*., optical tweezers operating at the 1064 nm wavelength cause an ca. 1.2°C increase in temperature for every 100 mW of power applied [26,27]. The optical tweezers in this study operate with a 1064 nm wavelength and the measured power at the trap focus was approximately 25 mW. The trap power was measured by manually positioning a detector (P/N: S121C, Thorlabs) at the exit aperture of the microscope objective. We moved the detector up and down by ca. 1–2 cm and found that the measured power did not undergo any appreciable change, thus ruling out the possibility that the angular spread of the beam would somehow lead to an underestimate of the laser power. Finally, we note that the laser power in the trap is less than this measured amount due to absorption by the immersion oil, glass and aqueous solution although this absorption should be negligible. Most cells are held in this trap for approximately 10–15 seconds. Thus, the maximum temperature change we expect the cells to experience is approximately 0.3°C, which makes DNA damage from heat unlikely, and also eliminates the possibility of creating convective flows in the sample that would disrupt the trapping efficacy (see video hyperlink). *Mohanty et al*. explored the extent of DNA damage caused by optical tweezers using a comet assay, where NC37 lymphoblasts were captured using wavelengths from 750–1640 nm at various laser intensities and exposure times [16]. On average, they had found that approximately 12% of DNA exposed to 120 mW of power at a wavelength of 1064 nm for 30 seconds was found to be degraded. It is estimated that the current study uses about 1/5 this power and 1/2 the exposure time. Presuming the amount of DNA damage and the energy input into the cell follow a linear dependency, the current study exposes about 1/10 the amount of energy that Mohanty and colleagues had dispensed. Consequently, we could reasonably estimate the amount of DNA damage inflicted using this method to be insignificant for forensic applications. Further, in the current study, neither the degradation index nor interlocus peak height ratios of the profiles, both of which are commonly used as measures of DNA fragmentation in forensic STR analysis, indicated significant levels of degradation in the samples.

## Conclusions

The objectives of this study were to optimize sperm cell collection for maximum DNA yield and to determine the number of tweezed sperm cells necessary to produce a complete STR profile using conventional DNA analysis methods. Additionally, we sought to explore how effective optical tweezers could be in separating sperm cells from a mixed sperm:epithelial sample such as is commonly seen in sexual assault evidence.

Optical tweezers offer several advantages when compared to other forensic cell separation methods explored. Unlike fluorescently-activated and magnetic cell sorting, optical tweezers can sort spermatozoa from a mixture with high specificity, as the handler can see the sperm cells at a microscopic level and thus choose only spermatozoa to be captured and transferred. Smaller numbers of sperm cells can also be isolated accurately using this method. Less than 60 sperm cells are needed to obtain a complete STR profile using optical tweezers whereas methods requiring antibodies have been shown to require approximately 10^5^ cells [10]. Optical tweezers also do not require the use of antibodies to successfully separate sperm and epithelial cells so complications regarding antibody binding specificity or efficiency do not exist. However, the optical tweezer equipment can be modified to view fluorescence (i.e. with an appropriate dichroic mirror, see for example [28–32]; consequently, sperm cell separation using antibody-based methods (such as that recently reported by Xu et al. [10]) could also be modified for use in optical trapping if needed, delivering cell separation not only by morphology but also antibody differentiation.

Laser capture microdissection is a microscopic method used for cell separation and is thus more similar to the optical tweezer method explored here. However, in the laser capture microdissection system, cells remain fixed on the microscope slide. In contrast, optical tweezers operate in an aqueous solution, and without the need for staining, fixing, or melting/catapulting cells to another surface. This provides more versatility during sperm cell isolation because spermatozoa can be lifted in the X, Y, and Z axes using the optical trap and maneuvered around other cells that are present in the mixture (as demonstrated in the aforementioned YouTube video link). Sample preparation is also easier and faster, which allows for a decreased overall sample processing time. Further, as optical trapping includes capture and movement of a specific target cell from an aqueous environment, the issue of accidental collection of nonsperm cells that may be clumped together with target sperm cells (a phenomenon commonly encountered during laser capture microdissection) is largely avoided. Lastly, when compared to its nearest competitor laser-capture microdissection (LMD), or the more recently described DEPArray system, the amount of time necessary to process a mixture sample is shortened dramatically using the optical tweezer method described herein. Sample preparation and optical trapping of 40–60 cells is routinely performed in our laboratory in less than 40 minutes, while the shortest analysis time from sample resuspension to LMD collection on a recent laser-capture microdissection report for a forensic sample was 143 minutes [33].Once the sexual assault cell sample is added to the cover slip positioned on top of the microscope slide, only approximately 45 minutes is needed to tweeze and collect ca. 50 spermatozoa for DNA extraction.

More importantly, effective cell separation at the front-end of the forensic DNA workflow could eliminate the need for complex back-end mixture deconvolution and interpretation protocols, which requires extensive additional training and validation, is somewhat subjective, and can take many hours of additional manual analysis time prior to case reporting [34]. Currently, laboratories do not have an effective solution for cell separation, relying on differential lysis procedures, which require the use of hazardous organic solvents and tedious manual pipetting processes. These issues add additional variation to a procedure that does not guarantee optimal cell separation and often still results in a need for mixture deconvolution and interpretation, leading to significant analysis backlogs for sexual assault cases [5,34].

This study effectively demonstrates that optical tweezers have the potential to be introduced as a useful tool for forensic applications, particularly as a method for sperm cell isolation and transfer prior to routine DNA extraction and analysis. The apparatus requires minimal training in part because of the joystick controlled microscope stage. In our lab, undergraduate students typically require a few minutes of explanation along with 10–20 minutes of practice before they are proficient at trapping and isolating cells. The tweezer apparatus can also be optically isolated with blackout materials (i.e. BFP1, TB4, etc. Thorlabs) and appropriate eyewear (i.e. LG11, Thorlabs) can be used to address any possible safety concerns related to the tweezer setup. This technology could impact the way forensic casework is conducted by allowing forensic biologists to disentangle cells from more than one contributor (i.e. sperm cells from vaginal epithelial cells (see hyperlinked video)) at the beginning of the DNA analysis workflow, allowing for a notable reduction in the current back-end bottleneck due to the labor-intensive nature of mixture interpretation.

## Supporting information

S1 Data Raw quantification and CE data. (XLSX)
